# Enhanced virtual microscopy for collaborative education

**DOI:** 10.1186/1472-6920-11-4

**Published:** 2011-01-26

**Authors:** Marc M Triola, William J Holloway

**Affiliations:** 1 Division of Educational Informatics, New York University School of Medicine, New York, New York, USA

## Abstract

**Background:**

Curricular reform efforts and a desire to use novel educational strategies that foster student collaboration are challenging the traditional microscope-based teaching of histology. Computer-based histology teaching tools and Virtual Microscopes (VM), computer-based digital slide viewers, have been shown to be effective and efficient educational strategies. We developed an open-source VM system based on the Google Maps engine to transform our histology education and introduce new teaching methods. This VM allows students and faculty to collaboratively create content, annotate slides with markers, and it is enhanced with social networking features to give the community of learners more control over the system.

**Results:**

We currently have 1,037 slides in our VM system comprised of 39,386,941 individual JPEG files that take up 349 gigabytes of server storage space. Of those slides 682 are for general teaching and available to our students and the public; the remaining 355 slides are used for practical exams and have restricted access. The system has seen extensive use with 289,352 unique slide views to date. Students viewed an average of 56.3 slides per month during the histology course and accessed the system at all hours of the day. Of the 621 annotations added to 126 slides 26.2% were added by faculty and 73.8% by students. The use of the VM system reduced the amount of time faculty spent administering the course by 210 hours, but did not reduce the number of laboratory sessions or the number of required faculty. Laboratory sessions were reduced from three hours to two hours each due to the efficiencies in the workflow of the VM system.

**Conclusions:**

Our virtual microscope system has been an effective solution to the challenges facing traditional histopathology laboratories and the novel needs of our revised curriculum. The web-based system allowed us to empower learners to have greater control over their content, as well as the ability to work together in collaborative groups. The VM system saved faculty time and there was no significant difference in student performance on an identical practical exam before and after its adoption. We have made the source code of our VM freely available and encourage use of the publically available slides on our website.

## Background

Traditional education of medical students in histology and pathology has long involved the use of microscopes in faculty-led laboratory sessions. This model is facing contemporary challenges such as curricular reform projects that have reduced or altered the timing and availability of microscope laboratory sessions [1], a lack of available space and equipment [2], and a move towards new teaching methods that include team-based learning and working in small groups[3,4]. Accompanying the structural curricular changes is a push to integrate teaching of physiologic and anatomic concepts and a competency-based education model [1,4,5]. These pedagogical approaches emphasize the interpretation of histology images and identification of functional structures over manual skills of using physical microscopes [6].

A growing trend across medical education is to use computer-assisted instruction to enhance or replace traditional teaching strategies and address many new pragmatic and pedagogical challenges like those listed above [7,8]. These approaches have particular promise in highly visual topics like histopathology [1,4,9-12]. Virtual Microscopes (VM) are computer-based programs that enable viewing, navigating, and annotating digital slides acquired from a camera-equipped microscope or a commercial digital slide scanning system. With the introduction of robust commercial systems, their use has been increasing throughout health professions education [2,7,13].

VM systems have many benefits for learners. Like other computer-based educational technologies, they have ubiquitous availability; excellent slides or rare samples can be digitized just once and then made available to large audiences simultaneously. Students can quickly and easily compare normal and abnormal, even on the same screen. Lacking are the mechanical barriers to the actual learning of histology such as focus, staining, lighting, etc. VM systems foster collaborative and team-based learning with students and faculty viewing or annotating together in ways impossible with traditional microscopes. Digital slides are searchable and can be automatically indexed into dynamic collections [11,13]. Faculty teaching with VM systems also experience advantages since they can pre-annotate slides outside of the lab or embed slides or links to specific views in other digital teaching materials [14].

VM applications have been adopted across a variety of health professions education programs including medicine, dentistry, and veterinary sciences [2,7,15-19]. Prior implementations have reported rapid and dramatic adoption of VM systems over physical microscopes [14,20]. Previous comparative evaluations of VM and traditional microscopy found equal satisfaction in quality of image and ease of use, and greater satisfaction with efficiency of learning and accessibility [1,4,14,16]. Scores on practical exams have not been negatively impacted by using the VM approach and in some cases have shown improvements [3,4,11,14,19,21]. These findings appear to be consistent across several different types of health professions students [15,16,19].

In the context of a curricular reform effort at our medical school, we re-evaluated the teaching of histopathology in the pre-clinical years. Building on the successes of previous implementations and best practices from the literature, we sought a VM solution that would empower our learners to have more control, improve access to teaching materials, and overcome the challenges to traditional laboratory-based teaching. Such a system would support collaborative content creation and annotation features for faculty and students. We also planned to implement new team-based learning sessions where groups of students would collaborate on tissue identification and structure location within a single slide. After a thorough evaluation of several commercial software solutions, we chose to build a standards-based open-source VM application designed from the ground up for collaborative teaching and learning.

## Implementation

We sought a solution that had fast performance, required no special software or plug-ins, and could be extended with custom functionality to support our desired learning and collaboration features. We chose the Google Maps Javascript Application Programming Interface (API, Google Inc., Mountain View California) for its speed, capabilities to handle immensely large image data sets, and familiarity to our students and faculty [22]. Free for academic use, The API provides a number of services to create interactive applications and annotations with user-created 'markers'. We created two components: a script to convert images produced by commercial slide scanners into the Google Maps format, and a web-based viewer application. To our knowledge this is the first implementation of VM technology using the Google Maps engine and one that overcomes many of the performance and system barriers of previous systems.

### Image Processing Script

Our tiling script converts digitized microscope slides produced by commercial slide scanner machines, generally Tagged Image File Format images, into 256 × 256 pixel JPEG tiles suitable for use within a Google Maps API web-based viewer. The script generates pyramidal sets of tiles for each "zoom level" in the Google viewer. The source images range in size from several hundred megabytes to several gigabytes depending on the scanned magnification and the size of the tissue sample. The script will convert a typical 40 × scanned slide file to approximately 45,000 individual map tiles. For 100 × slides the tile set approaches 200,000 files. The resulting image tile set is a simple series of static files and can be hosted on any web server or content distribution network. This approach decouples our slide scanning digitization process from the viewer application and allows us to include slides digitized by a number of commercial vendor systems.

### Instructional Design of the VM Viewer

We put significant development effort into the digital slide viewing system to make it as easy to use and as broadly accessible as possible. Our goal was to empower all users of the system, faculty and students alike, to annotate and create content. Since we are using the familiar, easy to use Google Maps engine, our system works on virtually all recent browsers under any operating system as well as mobile devices such as the iPad.

**Figure 1:**
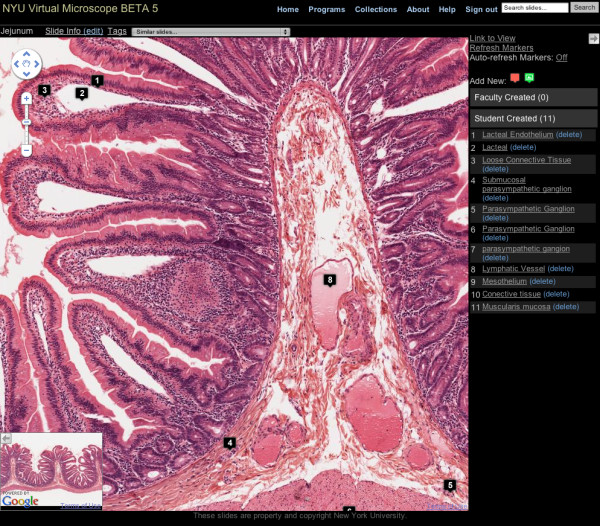
**Virtual Microscope slide viewing screen**.

The slide viewing screen is shown in Figure 1. The window consists of a main viewing area with a mini-map to provide a navigational overview. The toolbar at the top allows faculty to edit the slide's descriptive data, any user to tag the slide, and add quick links to other slides in our system. There is a sidebar that lists the markers for that slide and has icons to add new markers. Clicking on either the marker title in the sidebar or the marker icon on the slide itself will center the marker on the map and zoom the image to the level the marker's author chose when creating that annotation. The mini-map and sidebar can be collapsed so that almost the entire screen is used for slide viewing. When viewing a slide, the user can also selectively hide the marker icons on the slide.

### Slide Annotation

**Figure 2:**
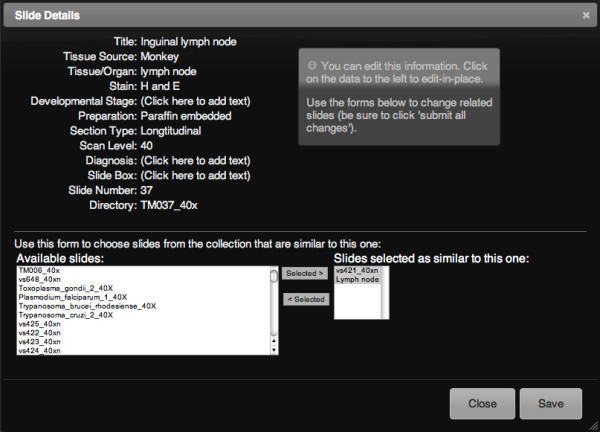
**Faculty slide meta-data entry screen**.

The system includes several features to annotate slides with both markers (visual signposts placed on the slide itself) and with extensive meta-data. Faculty can add meta-data to every slide in the following categories: source organism, tissue/organ type, stain, developmental stage, preparation, section type, scan level, and diagnosis (Figure 2). All of these data are searchable so that faculty and students can quickly find all slides of a given tissue type or stain for example. The system also has a 'similar slides' feature where faculty can attach links to other slides and students can easily see a different example of the same tissue or structure.

Our VM also permits students and faculty to add arbitrary free-text tags to any slide. This approach allows the entire educational community at our school to add additional searchable terms, create ad hoc or informal collections of slides, or to organize slides in unanticipated ways. The tags collectively create a 'folksonomy' [23] that reflects informal classifications by users that complement the formal terms and meta-data added by course faculty. These tag terms are weighted into a searchable 'tag cloud' that shows relative prominence of terms across the entire system.

**Figure 3:**
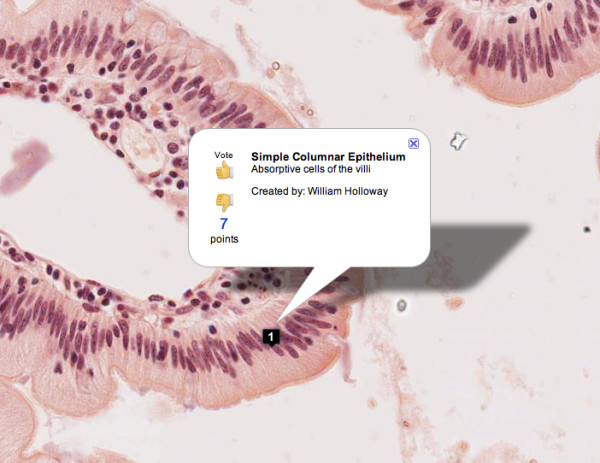
**Student marker annotation**.

The VM has extensive support for markers in the form of small icons placed on the map by users. Any user can create markers with faculty markers being visually distinct from student markers. There are two types of markers supported: pushpin markers and image markers. Pushpin markers have a title and description and allow the user to precisely point out a structure. When clicked, the marker will be centered the screen and the image will automatically zoom to the level at which the marker was created (Figure 3). Image markers allow users to embed sequences of images with captions directly in the context of the cellular structures. These short, embedded learning sequences can support images of gross anatomy, diagrams of signal pathways or physiologic processes, or even images of PowerPoint slides with captions. The "Auto-refresh Markers" feature allows the annotation data to be automatically synchronized across web-browsers, which is particularly useful during real-time collaborative laboratory sessions. Each time the course is offered, markers from the previous year's students are removed from the system so that the slides can be presented anew as "unknowns."

A social networking approach was used to give the community of learners and faculty a means to peer review slide annotations and collectively promote or demote markers based on their perceived value. Any student or faculty can vote on any marker with a "thumbs up" or "thumbs down" to add or subtract to its total score. The relative score each marker has determines its priority in the marker list. If a marker receives a cumulative vote score of less than -5, it is automatically removed from the system and only visible to its author.

### Practical Examinations

The VM system has a practical exam mode that includes several hundred slides only visible during exams. Faculty are required to peer review and approve the image quality of slides prior to them being included in an exam. The VM exams are administered on student laptops in the laboratory using our school's wireless network. Exam security is achieved using a combination of our student honor code and faculty proctors in each room. Since each student is working on his or her laptop during exams, they can self-pace and revisit slides they are unsure of. This change is a significant enhancement over traditional microscope or kodachrome-based exams that forced students into a timed lockstep for each question. An additional benefit to the VM-based exam is that we can randomize which slides students see, with different students getting different examples of the same tissues on each exam. Faculty grading of the exam is now done electronically and the VM has several tools to make the grading process much easier and faster than paper-based handwritten answers. These features include the presentation of the 'correct' answer in context with the typed student response, a single click to give full or partial credit, and a performance report that can be imported into our learning management system for automated grade delivery to students.

### System Pilot

We implemented the system as a pilot in December of 2008 alongside our traditional microscopes. Students were able to bring their laptops to the laboratory and use the VM at the same bench with their individual microscopes. The VM was introduced as an optional supplemental resource to an entire class of students during the pilot period. Accompanying the introduction of the VM was a change in laboratory structure from students working individually to their working in small groups to collaboratively identify structures and tissues. Faculty were given live training sessions in the use of the system as well as online screencasts with step-by-step instructions. Students were given online help text and screencast training. Reports by course directors and our evaluation of usage data showed rapid adoption of this system by students at the expense of their using the physical microscopes. After one semester of piloting this system, the school chose to abandon the use of traditional microscopes in favor of our VM system.

## Results

We currently have 1,037 slides in our VM system. These slides are comprised of 39,386,941 individual files that take up 349 gigabytes of server storage space. Of those slides 682 are for general teaching and available to the world as well as our students; the remaining 355 slides are used for practical exams and have restricted access. The teaching slides are in six core topic areas: Electron Microscopy (n = 52), Hematology (n = 22), General Histology (n = 436), Neuroscience (n = 15), Parasitology (n = 71), and General Pathology (n = 85).

**Figure 4:**
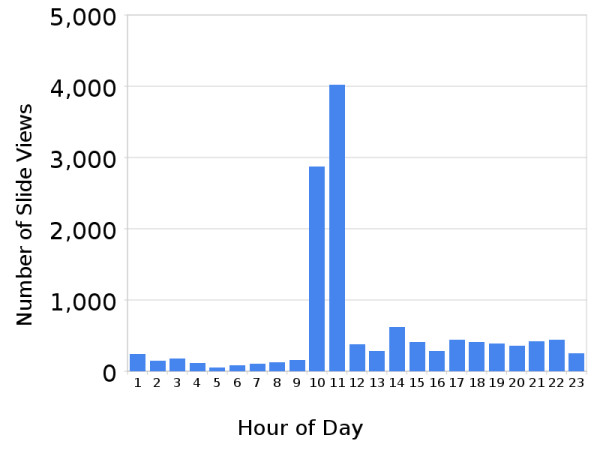
**Student slide views according to hour of day**.

The system has seen extensive use with 289,352 unique slide views to-date. We performed detailed analysis of usage during the most recent four-week period in which first year medical students were taking a general histology course. There were 12,982 slide views by all 164 students during the month, with a mean per student of 56.3. (SD = 34.64, min = 32, max = 225). The time of day that students viewed the slides is displayed in Figure 4. 6,896 (53%) of the views took place during laboratory hours (10 am-12 pm) and 6,086 (47%) took place outside of the lab with a majority (77.3%) of non-lab use between 4 pm and 12 am. Students used the VM system every hour of the day during the observation period. During the two-hour laboratory sessions, students on average viewed 21 digital slides and spent 9 minutes and 8 seconds per slide. During non-lab time, when students had greater control over their pacing, students looked at an average of 10 slides per hour and spent 10 minutes and 11 seconds per slide (P = 0.07 for the difference between in-lab and out-of-lab view duration). The most frequently viewed slides are listed in Table 1 and correlate with the course syllabus and laboratory session topics.

**Table 1:** Most frequently viewed slides (n = number of student views)

During laboratory hours:	During non-laboratory hours:
Spleen, small intestine, trachea, submandibular gland, tongue (n = 356)	Spleen, small intestine, trachea, submandibular gland, tongue (n = 126)

Trachea (n = 267)	Thick skin, pacinian corpuscle (n = 115)

Mesentary whole-mount (n = 259)	Lip (n = 107)

Artery, vein, nerve (n = 229)	Mesentary whole-mount (n = 99)

Kidney, radial section (n = 209)	Palmar skin (n = 98)

Jejunum (n = 203)	Trachea (n = 88)

Kidney (n = 196)	Scalp (n = 84)

Jejunum (n = 173)	Soft palate (n = 83)

Lip (n = 170)	Endochondral bone formation (n = 82)

Lung root (n = 154)	Lung root (n = 80)

During the histology course there were 621 annotation markers added to 126 slides (min = 1, max = 36 per individual slide). Of those 163 (26.2%) were added by faculty and 458 (73.8%) by students. 71 (44%) students participated in the voting up or down of 191 unique slide markers. The mean number of votes per marker was 1.8 (SD = 1.46, min = 1, max = 12). Six markers received enough down votes from students to be automatically removed from the system. The subjects of the student annotations are detailed in Table 2, with multi-cellular structures and individual cells being the most common targets. Students also added 61 unique free-text tags to 50 slides.

**Table 2:** Subjects of student annotations, n (% of total markers)

Multi-cellular structure (i.e. nephron, capillary)	189 (41.2)
Individual Cell (i.e. eosinophil, melanocyte)	178 (38.9)

Tissue type (i.e. connective tissue, myocardium)	68 (14.8)

Sub-cellular structures (i.e. nuclei)	4 (0.01)

Other/undetermined	19 (4.2)

The use of the VM system reduced the amount of time faculty spent administering the course, but did not reduce the number of laboratory sessions or the number of required faculty. Due to the efficiencies and workflow of the VM system, laboratory sessions were reduced from three hours to two hours without reducing the number of slides taught in each session. The course's 15 laboratory sessions are each taught by 14 faculty preceptors overseeing groups of students. The laboratory duration reduced the overall precepting time from 630 to 420 hours, saving 210 hours per year in faculty time and freeing up an additional 15 hours of laboratory space for other courses. Though we did not formally assess the workflow changes that enabled the reduction in laboratory session duration, reports from faculty credited the continuous access to all specimens and the students not having to share slide boxes and wait before they could use a given slide.

Student performance on the final summative practical exam was compared from one year prior to, and one year after the transition from microscopes to the VM. The 2007 pre-VM summative exam was administered to 165 students using traditional microscopes and had a mean score of 80.1 (SD = 5.38). The identical exam was administered in 2009 to 164 students using the VM and had a mean of 81.8 (SD = 11.9). There was no significant difference between the exam scores across the two modalities (t: -1.69, P-Value = 0.093, 95% CI = -3.73 < μ1-μ2 < 0.289).

## Discussion

Virtual microscopy has effectively replaced physical microscopy for our histology education and enabled us to implement new collaborative teaching and learning strategies. Students created the majority of markers and also participated in the voting system to peer-review quality and relevance within the system. This transition was not associated with any negative impact on a single comparison of identical practical exam performance, a finding that is similar to other studies [3,4,11,14,19,21].

The VM system also allowed us to change the structure of all histology laboratory sessions to team-based learning, with students identifying markers on the same slide at the same time in collaborative groups. For the first time, our students and faculty can truly work together around a single microscopic slide image. Evaluation of the student usage data showed that almost half was outside of the laboratory and occurred at all hours of the day and night, reinforcing the benefits of anytime/where computer-based learning resources. These types of returns on investment are some of the critical benefits of computer-assisted instruction [8].

One consequence of converting to solely VM-based education is the loss of training in the manual skills of microscopy. This is a controversial topic to many schools that recognize students may have the opportunity to use light microscopes in settings outside of the histopathology course, such as clerkship rotations or research projects [21]. Recent surveys of practicing physicians in the United States reveal that the skills of microscopy are still perceived to be important in clinical practice [6]. Regardless of the balance of VM versus traditional light microscopy modalities, the overall trend across medical education is decreased histopathology laboratory time [7,24]. This implies that training in microscopy skills will be reduced regardless of the chosen viewing modality.

Our VM does have some limitations. We currently are only offering one focal plane per slide and any color correction of slides or stains must take place at the time of scanning. VM systems use digitized slides that offer a snapshot of the tissue at that time so dynamic microscopic processes such as the movement of cilia or stain changes with metabolism cannot be shown. Though our software and our slides are freely available, institutions wanting to scan their own slides will need access to a slide scanning machine or pay for a scanning service, which may present barriers to this approach [2]. The viewer application is also dependent on the continued availability of the Google Maps JavaScript API. If Google should remove access to this, the slide tiling portion of the VM would still produce standard JPEG image tile sets, which could be used in other large image set viewers.

Though there have been many successful VM systems described in the literature, this is the first solution utilizing the freely available and immensely powerful Google Maps engine. This technology has many potential uses in healthcare education, especially among those fields that use large visual data sets such as radiology, gross anatomy, dermatology, and others. Our system, which decouples the image processing from the viewer application, also has the advantage of being independent of the image source. It supports images from various digital slide scanners, standard graphics files, or potentially data from medical scanning and imaging equipment.

### Dissemination

The Virtual Microscope application source code is available under the open source MIT License at the link below. This software requires freely available web server and database software that will run under most server operating systems. We also welcome access to our slide collection both within our community and for educational use in general to any other interested medical school or training program. Note that the slides at our NYU School of Medicine VM can be viewed by the public but only allow editing and annotating slide content by currently enrolled students and faculty. Given that this is web-based, the only requirement of users is a fast Internet connection and a modern web browser, both of which are ubiquitous at schools of medicine.

## Conclusions

Our virtual microscope system has been an effective solution to the challenges facing traditional histopathology laboratories and the novel needs of our revised curriculum. The use of a web-based system empowered learners to have greater control over their content and work together in collaborative groups. The VM system saved faculty time and did not impact student performance on an identical practical exam. Our choice of the Google Maps engine has enabled us to develop a powerful and extensible system that supports a variety of digital images for education.

## Availability and requirements

• **Project name**: NYU School of Medicine Virtual Microscope

• **Project home page**: http://code.google.com/p/virtualmicroscope/

• **Operating system(s)**: Platform independent

• **Programming language**: Python

• **Other requirements**: Django, MySQL

• **License**: MIT License

• **Any restrictions to use by non-academics**: As per the MIT License

## Competing interests

The authors declare that they have no competing interests.

## Authors' contributions

MMT and WJH created the software and all aspects of the VM system. MMT performed the data and statistical analysis. Both MMT and WJH participated in the drafting of the manuscript. All authors read and approved the final manuscript.

## Authors' information

Dr. Triola is the Director of the Division of Educational Informatics at NYU School of Medicine (http://dei.med.nyu.edu). Mr. Holloway is the Lead Architect for the Division of Educational Informatics at NYU School of Medicine.

## Pre-publication history

The pre-publication history for this paper can be accessed here:

http://www.biomedcentral.com/1472-6920/11/4/prepub
